# Sex Differences in Age-Related Cardiovascular Mortality

**DOI:** 10.1371/journal.pone.0063347

**Published:** 2013-05-20

**Authors:** Tomi S. Mikkola, Mika Gissler, Marko Merikukka, Pauliina Tuomikoski, Olavi Ylikorkala

**Affiliations:** 1 Department of Obstetrics and Gynecology, Helsinki University Central Hospital, Helsinki, Finland; 2 Folkhälsan Research Center, Helsinki, Finland; 3 National Institute for Health and Welfare, Helsinki, Finland; 4 Nordic School of Public Health, Gothenburg, Sweden; 5 National Institute for Health and Welfare, Oulu, Finland; University Medical Center Utrecht, The Netherlands

## Abstract

**Introduction:**

Sex-related physiological differences result in different expressions of diseases for men and women. Data are contradicting regarding the increase in the female risk for cardiovascular disease (CVD) at mid-life. Thus, we studied possible sex differences in age-adjusted mortality for CVD and non-vascular diseases stratifying our findings by specific age groups.

**Methods:**

Over one million deaths (1 080 910) reported to the Finnish nationwide Causes of Death Register in 1986–2009 were analyzed. A total of 247 942 male deaths and 278 752 female deaths were of CVD origin, the remaining deaths were non-vascular. The annual mortality rates were calculated per 100 000 mid-year population, separately for men and women in 5-year age categories.

**Results:**

The age-standardized risk of death from CVD was 80% higher for men (442/100 000) than for women (246/100 000). After age 45–54 the male CVD mortality rate elevated parallel to the non-vascular mortality, whereas in women the CVD mortality elevated considerably more rapidly than the non-vascular mortality from age 60 years onwards.

**Conclusions:**

Heart disease mortality in men accelerates at a relatively young age, but in women the risk shows a steep increase at approximately 60 years of age. These data emphasize the need to identify and prevent risk factors for CVD, especially in women in their mid-life years.

## Introduction

Cardiovascular disease (CVD) is the leading cause of mortality in Western populations. It is also known that the life-long risk for CVD is higher for men than women [1], [2]. In the last few decades this difference has somewhat narrowed due to a decrease in male, and an increase in female risk [3]. Age is the most significant determinant for CVD in both sexes, but the data are not uniform as regards the sex differences in CVD risk by age. A large body of epidemiological evidence supports the concept that in postmenopausal years the female risk for CVD elevates more rapidly than among same-aged men [4]–[8] while equally strong data dispute this difference [9], [10]. These contradicting results may be partly explained by methodological differences, e.g. in statistical analyses used. Moreover, the picture is further blurred with data that early menopause, either spontaneous or artificial, is accompanied with an elevation in age-adjusted risk for CVD in most [11], but not all [12] studies.

Finland is known for the high rate of CVD mortality [13], and indeed, the age-standardized mortality from CVD is 62% higher for Finnish men (179 vs. 110 per 100 000 men) and 43% higher for Finnish women (80 vs. 56 women per 100 000 women) than the respective average of European Union [14]. The Finnish population is rather homogenous, as regards to race, standard of living and healthcare. Furthermore, a comprehensive and accurate register includes data for causes of death throughout the country. All this enabled us to explore the sex differences in annual age-adjusted mortality for CVD in Finland between years 1986–2009.

## Materials and Methods

A total of 1 080 910 deaths from diseases and medical conditions reported to the Finnish nationwide Causes of Death Register in 1986–2009 were analyzed (Table 1). This register is based on the law and diagnoses are imported from death certificate undersigned by a physician and double-checked by medical experts at regional level and at Statistics Finland. The register is accurate, and particularly so for deaths due to CVD, as shown before [15], [16]. It is noteworthy that if the cause of death is not fully verified by clinical history or pre-mortal findings from specific examinations (e.g. electrocardiograms, laboratory), an autopsy is advocated; this occurs in approximately 31% of all deaths [16]. We used the European causes-of-death shortlist produced by Eurostat (2012) to define causes-of-death [17]. We focused on deaths from vascular diseases classified into ICD-9 codes 390–459, and into ICD-10 codes as I00–99, further divided into ischemic heart diseases (ICD-9∶410–414 and ICD-10: I20–I25, cerebrovascular diseases (430–438 and I60–I69) and other, non-vascular heart diseases 420–423+425–429 and I30–I33+I39–I52). Deaths from diseases and medical conditions, which were unrelated to cardiovascular system, were considered as one single group of non-vascular deaths.

**Table 1:** Overall mortality among men and women, Finland 1986–2009.

	Men per 100 000			Women per 100 000			*p*	
	Deaths	Crude	Standardized	Deaths	Crude	Standardized	Crude	Standardized
Vascular diseases, total	247 942	412	614	278 752	441	385	0.022	<0.001
Cardiovascular diseases	181 117	301	442	178 014	282	246	0.120	<0.001
Cerebrovascular diseases	46 866	78	122	74 991	119	104	0.001	0.154
Other vascular diseases	19 959	33	51	25 747	41	36	0.549	0.243
Non-vascular diseases	270 237	449	646	283 979	449	401	0.993	<0.001
Total	518 179	862	1260	562 731	890	786	0.023	<0.001

Age-standardized according to Nordic Standard Population (18).

The annual mortality rates were calculated for each of the selected cause-of-death category per 100 000 mid-year population, separately for men and women in 5-year age categories. We used the Nordic Standard Population [18] for calculating age-standardized mortality rates for both sexes. Sex differences were tested by using the test for relative proportions. We calculated the gradient for logarithmic mortality rates in different mortality groups for both sexes, separately for age groups below and above 55–59 years. Moreover, the differences of age-related differences in mortality for CVD and non-vascular diseases in men and women were analyzed by using Joinpoint Regression Program Version 4.0.1. The statistical significance level was set at p<0.05.

## Results

The age-standardized risk for CVD death in men (442/100 000) was 79.6% higher than the respective risk in women (246/100 000) (Table 1). The male dominance in overall (Table 2), CVD (Table 3) and non-vascular (Table 4) mortality was seen in all adult age groups. The CVD mortality was highest (approximately 6-fold) between 45–59 years of age (Table 3). The male dominance diminished gradually with advancing age; the male risk was only 1.24 times higher in ages 85 years or more. The sex difference, however, remained statistically significant for all age groups above 50 years.

**Table 2:** Overall mortality among men and women by age, Finland 1986–2009.

	Men		Women			
Age groups	Deaths	per 100 000	Deaths	per 100 000	Male/Female-ratio	*p*
0	3 339	452	2 580	365	1,24	0,462
1–4	467	16	417	15	1,07	0,986
5–9	336	9	308	8	1,13	0,994
10–14	340	9	288	8	1,13	0,985
15–19	581	15	387	10	1,50	0,931
20–24	951	23	576	15	1,53	0,865
25–29	1 373	33	763	19	1,74	0,785
30–34	2 666	61	1 320	31	1,97	0,549
35–39	5 317	115	2 520	57	2,02	0,221
40–44	9 814	204	4 508	97	2,10	0,022
45–49	15 786	346	7 254	163	2,12	<0.001
50–54	23 716	566	10 662	256	2,21	<0.001
55–59	34 237	920	14 848	387	2,38	<0.001
60–64	46 614	1 494	21 638	629	2,38	<0.001
65–69	59 493	2 399	33 342	1 080	2,22	<0.001
70–74	73 984	3 866	54 184	1 948	1,98	<0.001
75–79	84 285	6 256	87 049	3 655	1,71	<0.001
80–84	76 932	10 016	117 249	6 819	1,47	<0.001
85–89	50 926	15 900	113 694	12 313	1,29	<0.001
90–94	21 740	24 653	67 163	20 946	1,18	<0.001
95–	5 282	37 602	21 981	33 203	1,13	<0.001

**Table 3:** Mortality from cardiovascular diseases among men and women by age, Finland 1986–2009.

	Men		Women			
Age groups	Deaths	per 100 000	Deaths	per 100 000	Male/Female-ratio	*p*
0	23	3	24	3	0,92	0,998
1–4	14	0	18	1	0,75	0,998
5–9	16	0	11	0	1,39	0,998
10–14	19	0	15	0	1,21	0,999
15–19	49	1	21	1	2,23	0,989
20–24	87	2	36	1	2,31	0,981
25–29	127	3	43	1	2,82	0,969
30–34	362	8	105	3	3,30	0,906
35–39	982	21	187	4	5,03	0,72
40–44	2 370	49	357	8	6,40	0,371
45–49	4 519	99	657	15	6,71	0,076
50–54	7 775	186	1 169	28	6,62	0,001
55–59	12 314	331	2 151	56	5,89	<0.001
60–64	17 652	566	4 408	128	4,41	<0.001
65–69	22 863	922	8 957	290	3,18	<0.001
70–74	27 214	1 422	16 801	604	2,35	<0.001
75–79	30 276	2 247	29 232	1 228	1,83	<0.001
80–84	27 321	3 557	41 357	2 405	1,48	<0.001
85–89	17 596	5 494	40 789	4 417	1,24	<0.001
90–94	7 629	8 651	23 881	7 448	1,16	<0.001
95–	1 909	13 590	7 795	11 775	1,15	<0.001

**Table 4:** Mortality from non-vascular diseases among men and women by age, Finland 1986–2009.

	Men		Women			
Age groups	Deaths	per 100 000	Deaths	per 100 000	Male/Female-ratio	*p*
0	3 309	448	2 552	361	1,24	0,463
1–4	450	15	393	14	1,07	0,982
5–9	314	8	287	8	1,00	0,994
10–14	304	8	262	7	1,14	0,988
15–19	505	13	335	9	1,44	0,939
20–24	807	20	476	12	1.67	0,88
25–29	1 136	27	630	16	1,69	0,821
30–34	2 059	47	1 086	26	1,81	0,667
35–39	3 800	82	2 037	46	1,78	0,446
40–44	6 503	135	3 641	79	1,71	0,223
45–49	9 799	215	5 856	131	1,64	0,079
50–54	13 844	331	8 397	201	1,65	0,008
55–59	18 754	504	11 146	291	1,73	<0.001
60–64	24 152	774	14 677	427	1,81	<0.001
65–69	29 705	1 198	19 693	638	1,88	<0.001
70–74	36 869	1 927	28 533	1 026	1,88	<0.001
75–79	41 579	3 086	41 514	1 743	1,77	<0.001
80–84	37 646	4 901	52 235	3 038	1,61	<0.001
85–89	25 326	7 907	49 810	5 394	1,47	<0.001
90–94	10 780	12 225	30 227	9 427	1,30	<0.001
95–	2 596	18 481	10 192	15 395	1,20	<0.001

**Figure 1:**
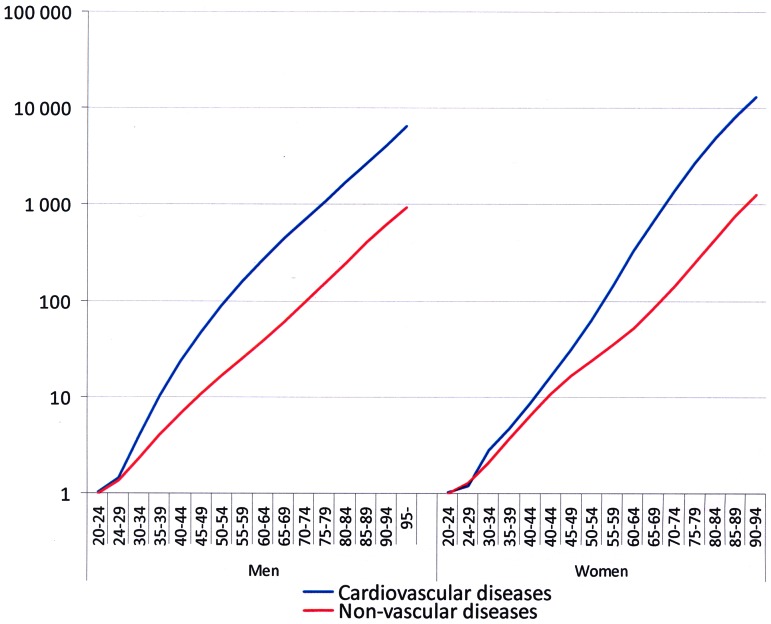
Mortality rates for cardiovascular diseases and non-vascular diseases in five-year age categories expressed on a logarithmic scale.

**Figure 2:**
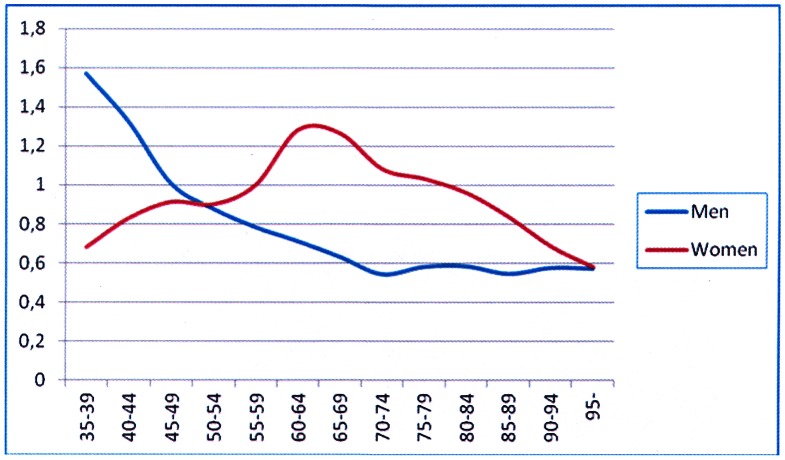
Male and female mortality for cardiovascular diseases in a given 5 year-period expressed as percentage increase from the mortality during preceding 5 years of age.

The CVD and non-vascular mortality curves expressed on a logarithmic scale for men were parallel (p = 0.065), whereas for women the CVD mortality showed a steeper increase (p<0.001). The CVD mortality in both sexes demonstrates significant changes at mid-life (Figures 1A and 1B). The increase in CVD mortality rate for men slow down after age of 40 years (Figure 1A), while for women the increase in the curve accelerates from age of 60 years and above (Figure 1B). Age groups 55 to 59 are the cutting points for sex-related CVD mortality; for men at ages 20 to 59 the slope for the logarithmic increase in risk of dying of CVD is 0.066 while at ages 55 and over the slope is 0.040 demonstrating a decrease in risk for CVD mortality. Instead, for women belonging to age groups 20 to 59 the slope for the logarithmic increase in the risk for CVD mortality is 0.053 while at ages 55 and over the slope is 0.058 demonstrating an increase in risk for CVD mortality. In contrast, the curves for non-vascular deaths are similar for both sexes (Figures 1A and 1B). A shift for female dominance is also seen when the CVD mortality in a given 5 year-periods is expressed as percentage increase from the mortality during preceding 5 years of age (Figure 2).

## Discussion

We analyzed more than one million deaths of which approximately one half were classified as CVD deaths. The data covered whole Finland during 24 consecutive years (1986–2009). It is evident from our data that the male age-adjusted CVD mortality is higher than the female mortality throughout life. This difference is most conspicuous at 40–44 years of age. However, after 55–59 years of age the female age-specific CVD mortality increases significantly more rapidly than the mortality for non-vascular causes, while in men, the CVD and non-vascular mortality curves are parallel. This results in a significant difference in the behavior of age-adjusted CVD mortality between men and women after 60–64 years of age.

The CVD mortality was much smaller in women than men of less than 45 years of age in Finland. These data are in line with previous ones from other countries [2]–[4]. This difference has been largely attributed to circulating estrogens present in women of fertile age. This explanation can be supported by epidemiological data that early menopause occurring spontaneously or as a result of bilateral oophorectomy is associated with 4–7–fold elevation in the risk for CVD [11]. However, other explanations for high male CVD mortality at young ages may well exist. It is a fact that various CVD risk factor, such as smoking, heavy drinking or poor dietary habits accumulate in young men, perhaps to the degree that CVD ensues [13]. It is also possible that various genes regulating e.g. insulin resistance and body fat distribution predispose preferably men to a risk for vascular disease [19], [20]. A sex difference may also exist in telomerase activity, which may endanger the tissue reparative capacity in men [21]. It remains open, however, why this defect should occur only in cardiovascular system and not in other organs; mortality for other than CVD causes showed only linear age- dependence.

There have been opposing interpretations whether CVD mortality is truly increased in women after menopause. Long-term follow-up data imply that menopause is followed by an increase in CVD mortality [4], [7], [22], [23], whereas some cross-sectional mortality data analyses dispute this change [9], [10], [24]. One possible explanation may be the overrepresentation of women in older age groups, which may exaggerate the mortality figures, especially if non-logarithmic calculations are employed. Furthermore, women live longer than men in western world, and therefore, eventually more women than men die of CVD [2], [25]. Our analyses indicate that age-standardized CVD mortality increases significantly after 60 years of age in women while the mortality for non-vascular diseases increases linearly with advancing age. We cannot deduce the reason(s) behind this phenomenon, but of course, menopause-induced hypoestrogenism is one possibility [26], [27]. If endogenous or exogenous estrogen mediates CVD protection it would be likely to be manifest at a young age and early menopause, but not anymore if initiated among women aged 60 years or more, as shown by the Women’s Health Initiative study [28]. In line with this assumption, we detected a significant elevation in the female CVD mortality at this age.

Our study has limitations. First, we do not have data on any other demographic characteristics than age and sex, and thus, we could not adjust mortality for factors, such as menopausal status, hormone and other therapy, socioeconomic status, smoking or body weight. We did the analysis separately for different periods of time and by five university hospital regions, and the results were similar to those shown here. Second, one can argue that CVD morbidity rather than mortality should have been studied. It is, however, known that CVD mortality closely reflects CVD morbidity [2], and therefore our data are likely representative for the general CVD occurrence. And finally, our analysis was conducted in racially homogenous Finnish population and thus, it is questionable how well our data can be applied to other populations with different ethnic backgrounds.

Our study also has several strengths. First, our data included over million deaths from consecutive 24-year period. Second, our Cause-of-Death Register covers the entire country and is accurate [29]. It should be noted that high standard health care provides accurate pre-mortal medical data, which facilitate reliable death diagnosis, and if not fully clear based on the pre-mortal data, autopsies are frequently carried out. And third, although CVD mortality in Finland has somewhat declined during the past decades [13], [30] it is still among the highest in Europe, and therefore, possible sex-specific differences in CVD mortality might be revealed in this population.

In summary, we show that there are sex-related differences in CVD mortality. While men have acceleration in heart diseases mortality at relatively young age, in women this risk shows a steep increase at approximately 60 years of age. This is likely a consequence of risk factors appearing some 10–20 years earlier. Therefore, it is highly important to improve CVD risk factors, in women already at fertile age as well as in their mid-life years.
